# Enhancing the Performance of Reversible Zn Deposition by Ultrathin Polyelectrolyte Coatings

**DOI:** 10.1021/acsami.3c14663

**Published:** 2023-12-02

**Authors:** Netta Bruchiel-Spanier, Omer Blumen, Linoy Lahav, Avigail Romem, Keren Shwartsman, Munseok S. Chae, Idan Bar-lev, Elad Gross, Netanel Shpigel, Daniel Sharon

**Affiliations:** † Institute of Chemistry, The Hebrew University of Jerusalem, Jerusalem 9190401, Israel; ‡ Department of Nanotechnology Engineering, Pukyong National University, Busan 48547, Republic of Korea; § Department of Chemical Sciences, Ariel University, Ariel 40700, Israel

**Keywords:** corrosion, polyelectrolyte, coating, zinc battery, PDDA, surface modification

## Abstract

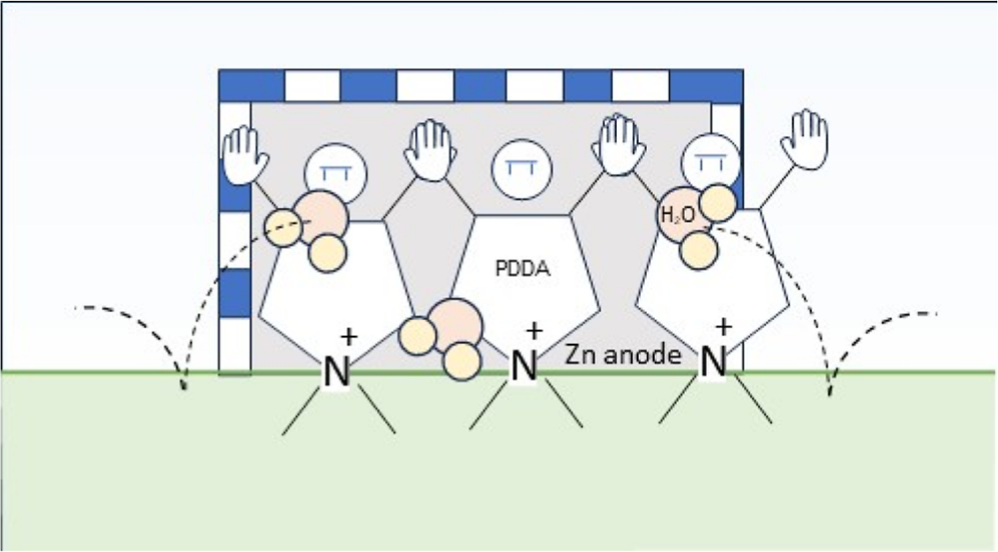

Modifying the surfaces of zinc and other metallic substrates is considered an effective strategy to enhance the reversibility of the zinc deposition and stripping processes. While a variety of surface modification strategies have been explored, their ability to be practically implemented is not always trivial due to the associated high costs and complexity of the proposed techniques. In this study, we showcase a straightforward method for preparing ultrathin polyelectrolyte coatings using polydiallyldimethylammonium chloride (PDDA) and polyethylenimine (PEI). The coatings, characterized by their electrostatic charge and hydrophobicity, suppress side reactions and even out the electrodeposition process across the substrate surface. The PDDA-coated anodes demonstrate significantly reduced voltage hysteresis, uniform zinc morphology, improved self-discharge rates, and an impressive Coulombic efficiency exceeding 99% over prolonged cycling. Our findings highlight the potential that such cost-effective and straightforward surface treatments could be widely applied in Zn metal-based batteries.

## Introduction

The increasing demand for sustainable energy solutions, such as solar and wind power, has driven extensive research in the field of energy storage technologies due to the need to address global energy challenges.^1^ Energy storage is a critical component in mitigating the intermittent nature of renewable energy sources and ensuring the reliability and stability of the electric grid.^2^ Among the various energy storage technologies, aqueous metal batteries have attracted significant attention due to their high safety, low cost, and environmental friendliness.^3^ Specifically, Zn-based batteries have emerged as promising candidates, offering a high energy density and potential for large-scale applications. While Zn-based batteries offer a high specific capacity that contributes to a high energy density and potential for large-scale applications, it is crucial to note that the practical energy density of aqueous Zn cells is also contingent upon the operating voltage and capacity delivered by the positive electrode.

However, Zn metal anodes face challenges related to their electrochemical performance and long-term stability. During the Zn deposition/stripping process, the competing hydrogen evolution reaction (HER) and dendritic growth leads to reduced Coulombic efficiency (CE), limited cycle life, and increased risk of localized corrosion.^4,5^ Consequently, there is a pressing need for innovative strategies to enhance the performance and stability of these batteries.^6^

Surface modification techniques have been widely studied to improve the performance and durability of Zn anodes. The reported approaches include the deposition of thin oxide layers, such as TiO_2_ or ZrO_2_, application of carbonaceous films, electroplating of various metals, and coating of polymeric films.^7−11^ Surface modification of Zn metal anodes can significantly reduce side reactions, regulate ion distribution, and provide physical protection layers.^12^ Yet, many of the proposed surface treatments involve utilization of costly compounds or involve complicated fabrication methods, thereby diminishing their practicality.

Polymeric coatings have emerged as a cost-effective strategy to enhance the stability of Zn electrodeposition. For instance, Cui et al. reported that a polyamide coating on Ti substrates could improve the average CE of Zn metal electrodeposition to 95%.^13^ Similarly, the use of a polyacrylamide/polyvinylpyrrolidone binary polymer blend as a coating on Ti substrates was found to increase CE to 98.4% after 300 cycles.^14^ Another noteworthy study demonstrated that a poly(vinylidene difluoride) coating enabled a symmetric Zn metal cell to achieve up to 2000 h of cycling operation and a rechargeable Zn metal-based battery to endure 4000 cycles.^15^ While these studies showcase the potential of various polymeric coatings, it is important to note that these methods can lead to several microns thick coatings, potentially resulting in increased cell resistance, uneven layer distribution, and compromised electrode adhesion.^16,17^ Considering the above, we demonstrate herein a facile and cost-effective approach to achieve highly reversible Zn deposition and stripping on the Cu substrate by employing ultrathin polymeric layers.

Polyelectrolytes are known for their remarkable ability to strongly adhere to surfaces, driven by electrostatic interactions, making them ideal choices for enhancing performance and stability in various applications.^18,19^ Leveraging their inherent self-assembling properties, strong adhesion to foreign substrates, and the formation of ultrathin layers, we selected to examine two positively charged polyelectrolytes: polydiallyldimethylammonium chloride (PDDA) and polyethylenimine (PEI). Electrostatically charged polymer coatings have demonstrated efficacy in resisting corrosion and stabilizing the electrodeposition of various metals.^20,21^ Consequently, we propose that ultrathin polyelectrolyte coatings with their distinctive attributes are particularly well-suited to meet the requirements of Zn metal anodes operating in aqueous solutions.

Our findings highlight the pivotal role played by the hydrophobic nature and interaction of the PDDA and PEI films with the electrolyte solutions, which significantly reduces parasitic reactions associated with H_2_ generation and formation of a stable Zn deposition layer. Our results demonstrate that Cu-PDDA-coated substrates exhibit a CE exceeding 99% and display high resistance to corrosion, underscoring the significant improvements achieved through nanoscale coatings on the overall performance of the Zn anode.

## Results and Discussion

The primary objective of this study is to explore the effect of self-assembled ultrathin polyelectrolyte layers on the electrode surface to enhance the reversible electrodeposition of Zn metal in mildly acidic solutions. For this purpose, we assembled PDDA and PEI polymers onto Cu foil and investigated the impact of relatively subtle physicochemical changes in the substrate interface. In aqueous solutions, PDDA is classified as a strong polyelectrolyte, while PEI is categorized as a weak polyelectrolyte. Both polymers tend to adsorb onto the substrate’s surface through electrostatic interactions.^22−24^ The polymeric coatings were obtained by immersing Cu foils in a 0.01 wt % polymer solution for 20 min under mild agitation. Subsequently, the Cu foils were rinsed in triple-distilled water to remove any residual polymeric chains.

Ellipsometry measurements of the polyelectrolytes on Cu substrates indicate that the thickness of the PEI and PDDA coatings are ≈4.25 ± 0.92 and ≈4.65 ± 0.22 nm, respectively.

**Figure 1:**
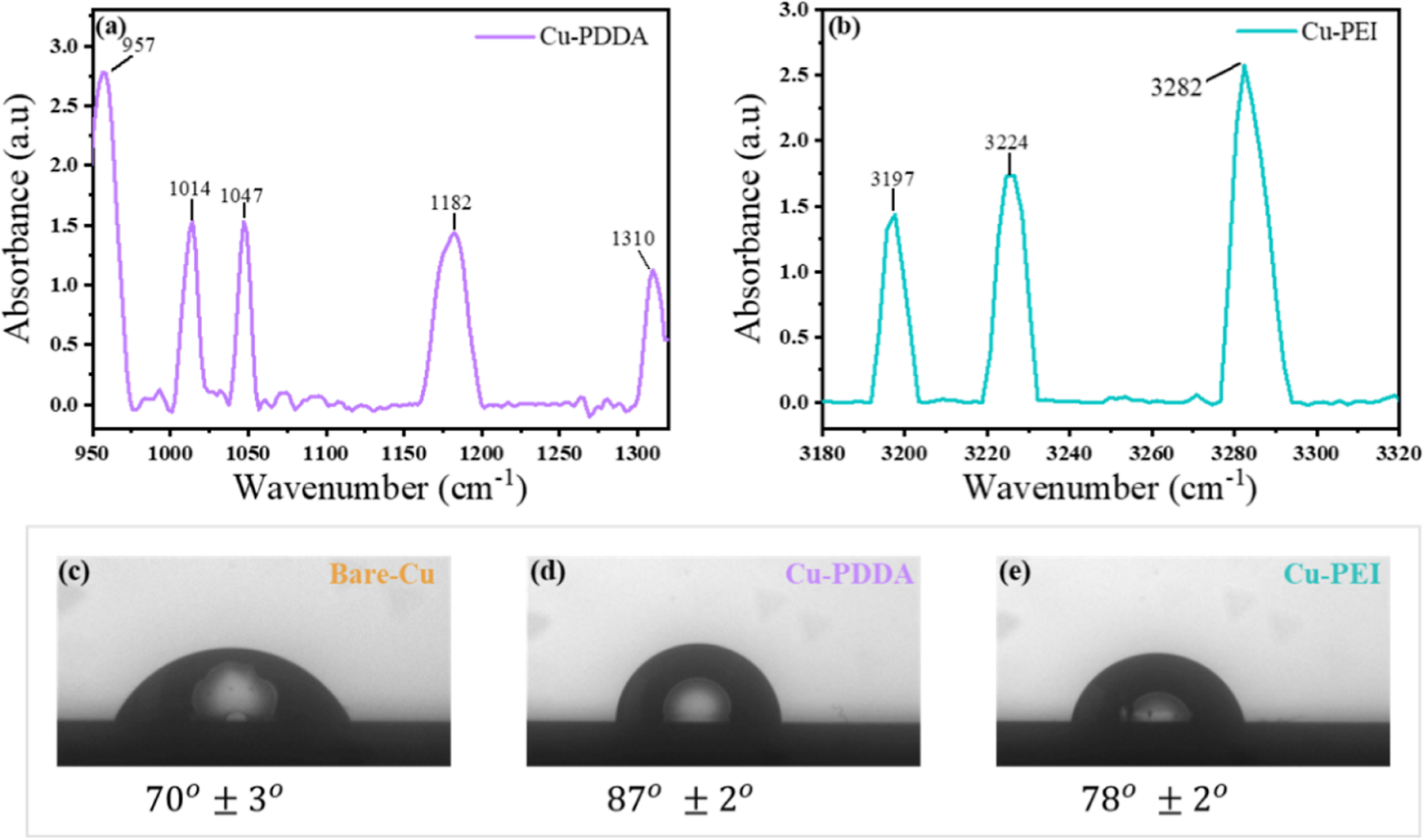
IRRAS spectra of (a) PDDA and (b) PEI adsorbed on a Cu substrate. Sessile waterdrop images and contact angle of (c) bare-Cu, (d) Cu-PDDA, and (e) Cu-PEI surfaces.

To validate and examine the chemistry of the ultrathin layers, infrared reflection-adsorption spectroscopy (IRRAS) measurements were applied. IRRAS is a versatile technique employed for investigating the IR properties of thin films adsorbed on reflective surfaces. As shown in Figure 1a,b, the PDDA-coated Cu showed characteristic spectra presenting pronounced peaks at 1014, 1047, 1187, and 1310 cm^–1^ corresponding to C–N stretching and bending vibrations. The peak at 957 cm^–1^ can be attributed to the stretching vibration of –(CH_2_) N^+^(CH_3_)_2_(CH_2_)–.^25^ In the case of the PEI coating, several peaks at 3197, 3224, and 3282 cm^–1^ were observed, which are associated with the stretching vibration of the N–H groups.^26^

To further characterize the surface properties, contact angle measurements were conducted. Figure 1c–e presents the contact angle of bare (bare-Cu) and coated Cu foil with PDDA (Cu-PDDA) and PEI (Cu-PEI) polymers, which were found to be 70° ± 2°, 78° ± 2°, and 78° ± 2°, respectively. Both polymers are more hydrophobic compared to bare-Cu. Yet, Cu-PDDA exhibits higher hydrophobicity than Cu-PEI which can be attributed to the more hydrophobic nature of PDDA due to its long hydrocarbon chains that repel water molecules. On the other hand, PEI has a highly branched structure with amine groups that can form hydrogen bonds with water molecules, making it more hydrophilic.^27^ However, when PEI is coated onto a copper surface, it may undergo chemical interactions or bonding with the surface, altering its properties and reducing the exposure of hydrophilic functional groups to water, which results in a decrease in the overall hydrophilicity of the surface.

**Figure 2:**
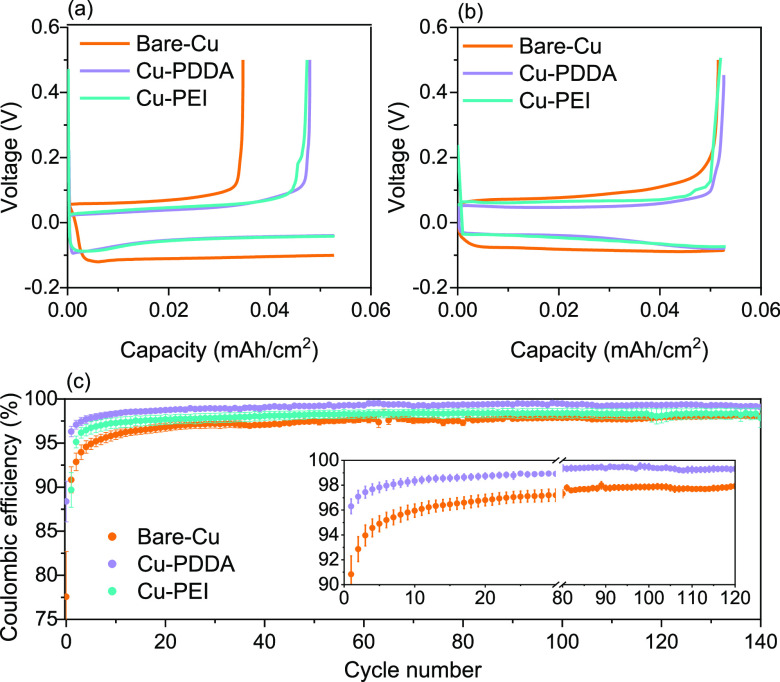
Deposition/stripping voltage profiles of the (a) 1st cycle and (b) 100th cycle for Zn in Cu||Zn cell at 0.5 mA/cm^2^ for 20 min, and (c) average CE values of Zn deposition/stripping on bare and coated Cu substrates.

The effect of the ultrathin layers on the Zn plating and stripping performance was evaluated by galvanostatic charge–discharge testing in asymmetric cells, utilizing Zn metal counter electrodes alongside coated and uncoated Cu working electrodes. The obtained galvanostatic charge–discharge profiles recorded at the first cycle and after 100 cycles at a current density of 0.5 mA/cm^2^ are shown in Figure 2a,b. The average CE is presented in Figure 2c. Based on the obtained data, two conclusions can be drawn—first, the coated Cu foils demonstrated substantially higher CE than the bare Cu foil. The latter exhibits an initial CE of 77.5%° ± 5% reaching a maximal value of 97.6%° ± 0.7% after ∼40 cycles. In contrast, the PDDA and the PEI-coated Cu present a high initial CE of 88.4%° ± 4% and 89.5%° ± 3%, respectively. Similar to the bare-Cu foils, stable CE was obtained after 40 cycles showing high values of 98.3%° ± 0.6% (PEI) and 99.5%° ± 5 (PDDA). It is important to emphasize that especially for reversible electrodeposition processes, the averaging of a large number of cells is highly important to avoid misinterpretation and inconsistent analysis due to the variation between different cells.^27^ Therefore, each point and its corresponding error bar represent the average of a minimum of four separate measurements conducted with the same coating.

The second noticeable effect relates to the voltage hysteresis between the plating and stripping processes. As can be seen, the overpotential required for the Zn deposition is larger for the bare-Cu substrate compared to the coated samples. Similarly, higher overpotential was required for the stripping process for the noncoated Cu. For all the samples, the voltage hysteresis decreased to some extent during the cycling due to stabilization of the electrode–electrolyte interface due to passivation and deactivation of reactive sites on the electrode surface. This improved kinetics implies that the nucleation and growth processes of Zn metal on the bare-Cu substrate require overcoming a larger activation energy barrier than the Cu-PDDA and the Cu-PEI substrates.^27^ The enhanced electrodeposition reversibility of Cu-PDDA remains consistent throughout all successive deposition and stripping cycles as at the 100th cycle (Figure 2b).

Several factors may contribute to the increased reversible Zn electrodeposition efficiency in the presence of polyelectrolyte coatings. One such factor is the surface charge of the polyelectrolyte layers when submerged in aqueous solutions. Both PEI and PDDA are considered charged polycationic layers when immersed in an aqueous solution. While the cationic character of PDDA is due to the presence of the positively charged ammonium groups, PEI undergoes protonation in water, even at relatively high pH values thus becoming positively charged.^28,29^ Charged polymeric coatings have been documented to interact with metal ions at the electrode surface, resulting in a more controlled and efficient electrodeposition process.^30,31^ It has been proposed that positively charged polymeric layers help distribute metal ions more evenly across the surface by electrostatically preventing the accumulation of high metal ion concentrations at heterogeneous regions on the electrode surface.^31,32^ Another possible contributing factor to the improved electrodeposition efficiency is the hydrophobic and electrostatically charged nature of both polyelectrolyte layers. These hydrophobic and charged coatings repel water molecules from the electrode surface.^33,34^ The simultaneous reduction of Zn^2+^ and water at the electrified interface leads to the local increase of the electrolyte pH, particularly in the SO_4_^2–^ environment, consequently leading to the formation of zinc hydroxide sulfate (ZHS) layers. Apart from their direct influence on the CE values, the presence of these insulating layers on the anode’s surface significantly affects the homogeneity of the applied electric field, which can result in nonuniform Zn growth and cell failure. The hydrophobic and electrostatically charged polymers repel water molecules, thereby suppressing the reduction of water on the electrode surface and improving its overall performance.^35,36^ As observed, the cells containing Cu-PDDA demonstrate significantly higher efficiency compared with Cu-PEI cells. The strong polyelectrolyte nature of PDDA, along with its hydrophobic and electrostatically charged properties in aqueous solutions, can explain its superior performance based on the two proposed mechanisms. Therefore, we conducted further investigations specifically focused on PDDA to gain a deeper understanding of the impact of polyelectrolytes on Zn electrodeposition performance when compared to that of uncoated Cu substrates.

Further analysis of the voltage profiles during the initial deposition step revealed a noticeable difference in the voltage slope between the bare and coated samples. The coated samples display a rapid transition into a plateau indicative of continuous Zn metal growth. On the other hand, in cells of bare-Cu substrates, the voltage profile during the early stages of the deposition process lacks an immediate potential drop. Instead, it demonstrates a gradual decline before eventually stabilizing. This observation may be attributed to the formation of an alloy between Zn and the Cu substrate before the subsequent deposition of pure Zn metal on top of it.

**Figure 3:**
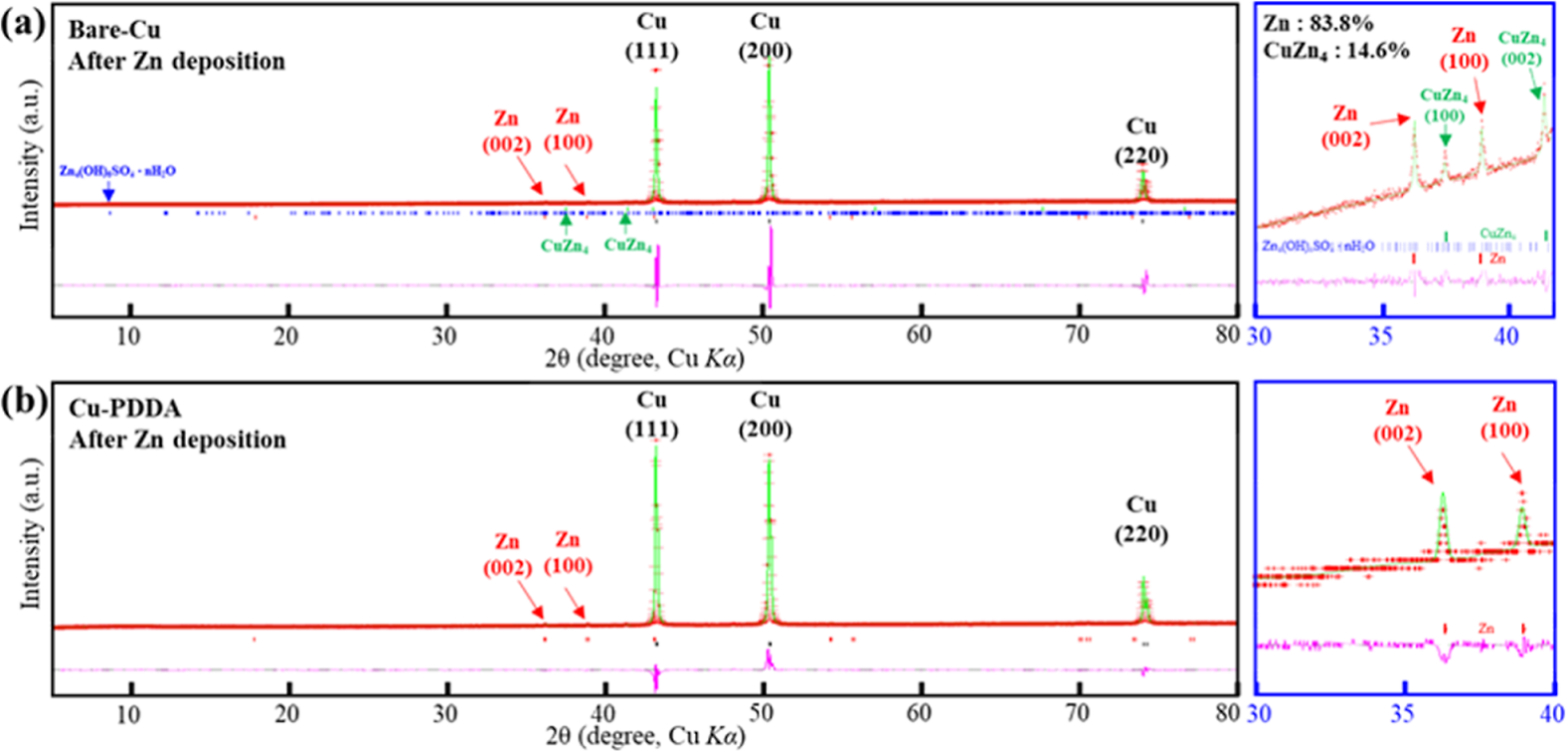
XRD patterns of (a) bare-Cu and (b) Cu-PDDA after the deposition of Zn at 0.5 mA/cm^2^ for 20 min. Green lines, calculated; red points, experimental; and pink lines, difference. Bragg positions: red bars, Zn; green bars, CuZn_4_; and blue bars, Zn_4_(OH)_6_SO_4_·*n*H_2_O.

X-ray diffraction (XRD) measurements provided additional evidence supporting the proposed electrodeposition mechanism on both coated and uncoated Cu substrates. As shown in Figure 3, characteristic diffraction peaks of metallic zinc, positioned at 36.3 and 39°, are observed in both bare-Cu and Cu-PDDA substrates after the initial deposition. However, only the bare-Cu substrate displayed two additional peaks at 37.5 and 41.4°, which are attributed to the presence of the CuZn_4_ alloy. The formation of CuZn_4_ alloy during the electrodeposition of Zn on Cu substrates has been previously reported.^37−39^ Furthermore, there was a notable disparity in the XRD intensity ratio between the Cu peaks (111) and (200). The bare-Cu samples exhibited a stronger (200) peak, suggesting the potential influence of the zinc alloying process. We propose that the initial low CE observed in bare-Cu during the first cycle can be ascribed to the irreversible formation of the CuZn_4_ alloy. The absence of an alloying process in the coated Cu anodes may result from the protective barrier of the ultrathin polymer coating on the Cu-PDDA substrate, which likely restricts direct interaction and diffusion of Zn into the Cu lattice. Furthermore, the applied coating layers impede zinc diffusion into copper, raising the activation energy barrier necessary for alloy formation. Concurrently, these layers may alter the electrode’s surface properties, lowering the energy barrier for zinc electrodeposition as can be seen from the lower deposition overpotential.

**Figure 4:**
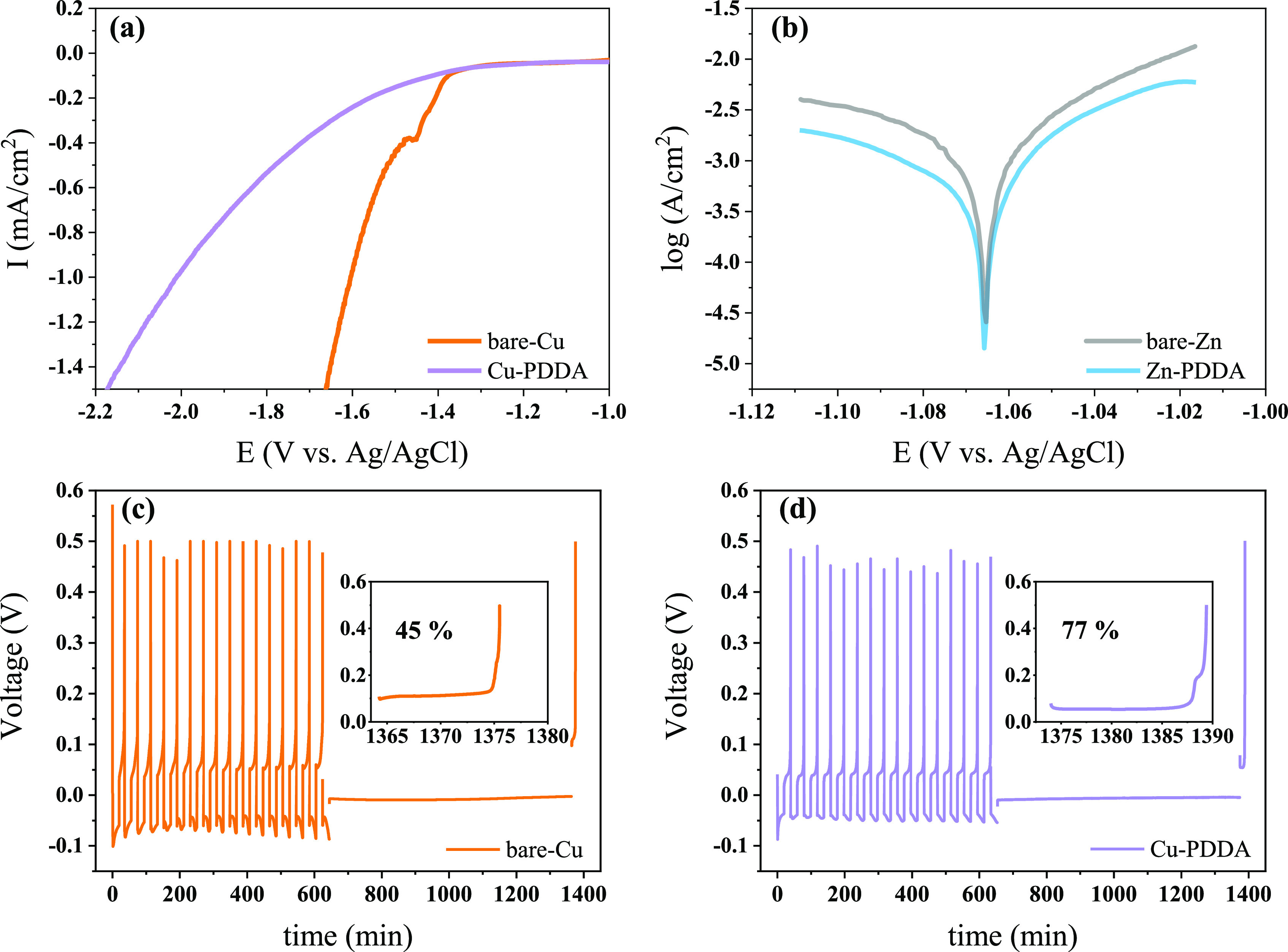
(a) Cathodic polarization analysis measured at 5 mV/s for bare-Cu and Cu-PDDA. (b) Tafel plots for bare and coated Zn substrates. Standing corrosion tests were performed for (c) bare-Cu and (d) Cu-PDDA substrates in 1 M ZnSO_4_ electrolyte solution.

To assess the protective efficacy of PDDA-coated substrates against HER, we performed linear scanning voltammetry as presented in Figure 4. The measurements were conducted in 1 M Na_2_SO_4_ to differentiate between the current of Zn ions reduction and the HER. The linear scanning voltammetry profiles reveal a markedly higher overpotential for water splitting on Cu-PDDA substrates compared to uncoated Cu, suggesting that the bare-Cu surface exhibits greater catalytic activity toward H_2_ evolution. We propose that the PDDA layer acts as a physical and chemical barrier to suppress the HER on the Cu substrate. This suppression is critical as the HER is a prevalent competitive side reaction during Zn reduction. Importantly, our data show that the PDDA layer does not hinder Zn reduction; in fact, it facilitates a lower overpotential for Zn deposition compared to the uncoated electrode (Figure 2a). We can see that PDDA not only does not act as a barrier to the Zn reduction reaction; rather, it promotes it in comparison to the uncoated electrode as evidenced by the low overpotential for Zn deposition (Figure 2a). The dual effect of promoting Zn deposition while mitigating the HER makes the PDDA coating an excellent candidate for enhancing the durability of Zn anodes.

Electrochemical impedance spectroscopy (EIS) measurements further substantiate the protective role of the PDDA layer on coated Cu-PDDA versus uncoated Cu substrates after electrodeposition (Figure S1). Initial EIS measurements yielded Nyquist plots, where the coated Cu-PDDA sample manifested an increased impedance semicircle diameter, which aligns with the expected electrochemical signature of an effective protective layer. Notably, after 10 electrodeposition cycles, a decrease in the impedance was observed for the PDDA-coated substrate. In contrast, the Nyquist plots of the uncoated Cu electrode displayed an increased impedance semicircle diameter over successive cycles. These observations suggest that the PDDA layer serves as a protective coating that after stabilization enables more effective electrodeposition processes.

Building on the insights gained from the suppression of the HER, we extended our exploration to examine the PDDA layer’s protective role against chemical corrosion. To this end, linear polarization experiments were performed to compare the corrosion behavior of uncoated (bare-Zn) and PDDA-coated (Zn-PDDA) Zn electrodes Figure 4b illustrates that the corrosion potential of both coated and uncoated Zn electrodes remains the same, while the corrosion current of Zn-PDDA is lower than that of bare-Zn. The higher corrosion current observed for the bare-Zn electrode indicates its greater susceptibility to corrosion compared to the Zn-PDDA coated electrode. The increased resistance to corrosion can be attributed to the decreased likelihood of localized corrosion on the Zn surface.^40^ The hydrophobic and positively charged PDDA coating may inhibit localized corrosion by providing a barrier that prevents the penetration of corrosive species such as dissolved oxygen and free water molecules into the metal surface.^27^ This suggests that the presence of the PDDA layer contributes to the stability and longer lifespan of the Zn metal electrode.

To assess the stability of the electrodeposited Zn metal during long-term exposure to corrosive environments, a standing corrosion test was employed to simulate the self-discharge conditions. The test includes 15 Zn deposition/stripping cycles, followed by an additional deposition process on both bare-Cu and Cu-PDDA surfaces. Subsequently, the substrates were left at an open circuit potential for 12 h before being stripped. The CE was calculated by comparing the amount of Zn stripped after the 12 h period to the predeposited amount (Figure 4c,d). The results revealed that bare-Cu exhibited poor corrosion resistance with a CE of only 45%, in contrast to the higher CE of 77% achieved for the Cu-PDDA substrate. This experimental finding indicates that PDDA continues to function as a protective layer after electrodeposition on the coated Cu foil. This evidence reinforces the premise that PDDA not only suppresses hydrogen evolution on the Cu substrate but also protects the electrodeposited Zn metal from corrosive degradation, thereby ensuring a more stable and efficient electrodeposition process.

**Figure 5:**
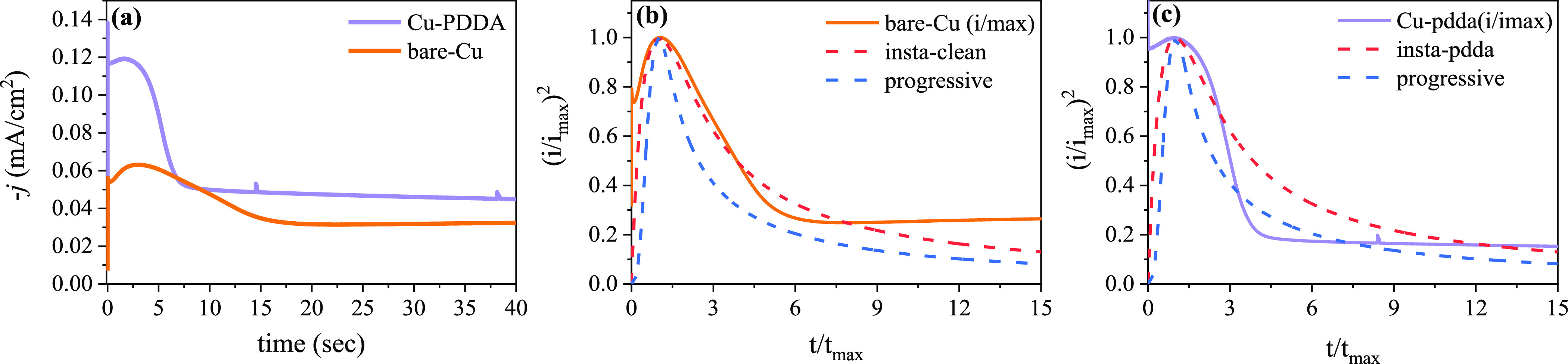
(a) Current–time transient resulting from chronoamperometry experiment for the deposition of Zn on Cu and Cu-PDDA substrates at a fixed potential of −0.6 V vs SCE. The nondimensional plots for (b) Cu and (c) Cu PDDA substrates.

To investigate the effect of the PDDA layer on the zinc metal nucleation and growth mechanism, we conducted transient chronoamperometry experiments of Zn electrodeposition on Cu-PDDA and uncoated bare-Cu substrates. Figure 5 shows the obtained current transient during Zn electrodeposition at a potential of −1.255 V (vs Ag/AgCl). The current response exhibits three distinct features corresponding to nucleation and growth during Zn electrodeposition.^41−43^ Initially, the current increases due to an increase in the active surface area of the electrode caused by the growth of Zn crystals. After a certain period (*t*_max_), the current density reaches its maximum value (*j*_max_) when the nucleation sites have grown sufficiently large, resulting in the overlapping of diffusion zones as predicted by the Avrami theorem. Subsequently, the current density decreases and stabilizes, in accordance with the controlled electrodeposition equation. Notably, the Cu-PDDA sample shows a higher *i*_max_ than the bare-Cu sample under the same applied potential, indicating a lower overpotential requirement for the electrodeposition of Zn on the Cu-PDDA substrate. Additionally, the current drops faster after *i*_max_ in the Cu-PDDA sample, suggesting differences in the nucleation and growth mechanisms between the two samples.

To gain further insights into the nucleation and growth mechanisms, we applied the Scharifker and Hills models.^44^ This theory describes nucleation on foreign substrates in terms of two limiting cases: instantaneous and progressive nucleation. In the instantaneous case, nucleation occurs rapidly compared with the rate of crystal growth, with nuclei forming at all possible growth sites within a very short time. In contrast, in the progressive case, nucleation occurs slowly and continues to take place at the surface while other clusters are growing. Figure 5b,c displays the experimental curves of the transient chronoamperometry experiments, plotted as normalized current density (*j*/*j*_max_)^2^ versus time (*t*/*t*_max_), and compares them to the proposed fitting models for instantaneous and progressive nucleation. The bare-Cu substrate initially correlated with the instantaneous growth; however, for extended reaction times, the measured current departs from the models and shows a gradual increase in the current rather than the expected stabilization. Previous research has suggested that the increase in current is caused by a gradual increase in the electrode’s effective surface area, which promotes more HER and other side reactions.^43,45,46^ This localized increase in surface area has also been linked to the formation of irregularly shaped metal structures during uncontrolled electrodeposition processes.

On the other hand, the Cu-PDDA substrate exhibits a more complex growth pattern. Initially, it also follows instantaneous growth, but beyond *t*_max_, it experiences a sudden transition toward more progressive growth behavior. This transition is accompanied by a drop in the normalized nucleation rate, indicating a shift toward more orderly growth. The current density levels off after a while, which suggests that there is a less HER on the Cu-PDDA substrates. This observation demonstrates that the PDDA layer significantly influences the electrodeposition mechanism, resulting in a more uniform zinc deposition which in turn enhances the stability and efficiency of the anode.

**Figure 6:**
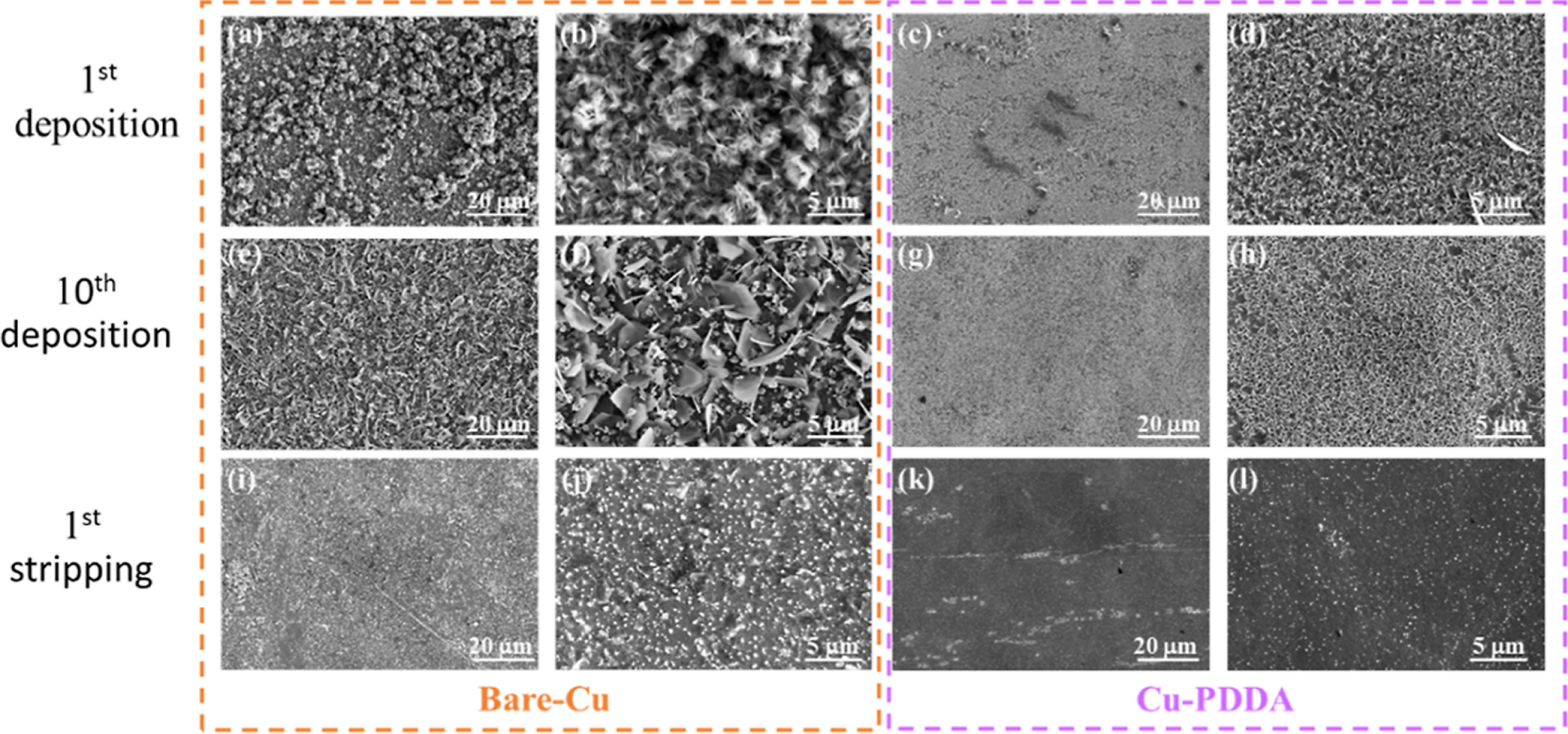
SEM images of bare and coated Cu after the (a–d) first deposition, the tenth deposition (e–h), and after the first stripping (i–l).

Scanning electron microscopy (SEM) imaging clearly reveals how the PDDA coating affects the zinc electrodeposition mechanism. Figure 6 displays the SEM images of the first and tenth deposition, as well as the first stripping processes, for both bare-Cu and Cu-PDDA substrates. The difference between the two substrates is highly noticeable. As seen, after the first deposition small aggregates of Zn were formed onto the bare-Cu substrate while a very homogeneous structure was achieved for the coated substrate with the coated substrate exhibited a very homogeneous morphology. This is consistent with the results of the transient current experiments (Figure 5), which indicate a more controlled and even growth process on the Cu-PDDA substrate. With increasing deposition/stripping cycles, the ordered structure on the Cu-PDDA surface is preserved, while the structure on the bare-Cu substrate becomes much more disordered (Figure 6e–h). Furthermore, complementary EDS analysis (Figures S2 and S3) reveals that after electrodeposition, the ratio of Zn/O is significantly higher in the Cu-PDDA-coated samples compared to the uncoated ones. This suggests a more efficient Zn electrodeposition on the Cu-PDDA substrate and the formation of a greater quantity of heterogeneous oxidized side products on the uncoated Cu substrate. These findings indicate that the PDDA coating effect on zinc nucleation and growth remains stable over multiple cycles, preserving the structural benefits imparted by the PDDA layer.

After the Zn stripping (Figure 6i–l), it is evident that substantial amount of residues persists on the bare-Cu surface, whereas the Cu-PDDA surface exhibits nearly complete removal of all of the products that were formed during the electrodeposition process. Residues observed on the Cu substrates could potentially include unoxidized Zn metal or byproducts like zinc hydroxy sulfates. Unoxidized Zn metal (“dead Zn”) can emerge when Zn fragments lose their electrical connectivity to the electrode surface. The irregular morphology of Zn deposition on the uncoated Cu surface may contribute to the generation of such disconnected fragments. Another plausible explanation for the enhanced stripping performance could be the contribution of positively charged PDDA chains, which assist in extracting Zn^2+^ ions from the surface.^47^ The complete removal of Zn enables the continuous formation of a homogeneous layer during each deposition. In contrast, the presence of residues on the bare-Cu surface leads to the formation of nonuniform and complex structures during subsequent deposition that damage the efficiency of the cell.

**Figure 7:**
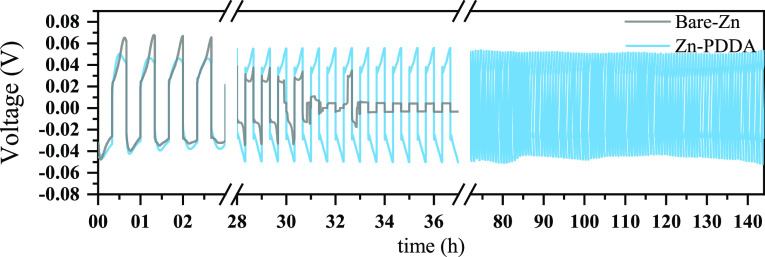
Deposition/stripping profiles of symmetric cells of bare-Zn and Zn-PDDA anodes at 0.5 mA/cm^2^.

Aside from influencing efficiency, the Zn deposit morphology can influence cell cycle life. Large vertical Zn crystals may penetrate through the separator and reach the counter electrode, causing a short circuit. To investigate cell stability during prolonged cycling, we compared the deposition and stripping profiles of symmetric bare-Zn and Zn-PDDA cells. As shown in Figure 7, the Zn-PDDA cell demonstrates stable polarization and superior durability compared to the bare-Zn symmetric cell. The bare-Zn cell exhibits larger polarization during the initial cycles, followed by rapid decline and limited lifetime, often resulting in a short circuit after only ∼30 cycles. Over time, the bare-Zn cell displayed a decrease in the polarization overpotential, attributed to the accumulation of soft short circuits.^35^ As mentioned above, the chemophysical properties of the PDDA coating promote uniform deposition, leading to a stable overpotential and extended lifetime of the cell. The enhanced cycling stability of the Zn-PDDA cells, while notable, does not yet fulfill the extensive longevity required for some applications. The significant enhancements provided by the ultrathin PDDA layer highlight the potential of carefully designed polyelectrolyte coatings to further prolong the anode’s operational durability.

The results clearly show that ultrathin polyelectrolyte films, just a few nanometers thick, significantly improve the electrodeposition behavior of Zn metal. Uncontrolled ion migration across the Cu surface in uncoated samples reduces Zn^2+^ ions to form unevenly distributed nuclei. These nuclei subsequently develop into nonorder Zn morphologies along thermodynamically favored sites, which is consistent with prior research utilizing ZnSO_4_ electrolytes in aqueous solutions. For the Cu-PDDA substrate, the presence of a positively charged polymer chain ensures a uniform and dense Zn-ion distribution on the surface. The PDDA chains may restrict the movement of ions and homogenize the Zn-ion flux by guiding the ionic transport process.^13,43,48,49^ Furthermore, the homogeneous positive charge of PDDA contributes to a uniform charge distribution across the surface, thereby promoting uniform growth of the deposited Zn on the coated Cu substrate.^47,50^ Additionally, a decreased competing HER during electrodeposition on the Cu-PDDA substrate can also contribute to improved homogeneity. The collective effects of the PDDA layer result in a markedly improved and stable zinc metal electrodeposition process.

## Conclusions

This study systematically examined the impact of a self-assembled ultrathin polyelectrolyte coating on the efficiency and stability of reversible Zn deposition in mildly acidic solution. When compared to uncoated electrodes, we found that positively charged PDDA polyelectrolyte coatings greatly increased Zn electrodeposition efficiency and stability. The PDDA layer acts as a barrier against the HER on the copper substrate, which is a critical aspect for advancing Zn metal electrodeposition. Additionally, Zn deposited on PDDA-coated copper exhibits enhanced resistance to corrosion, likely due to the hydrophobic and charged characteristics of PDDA that repel corrosive agents and block their access to the underlying metal. Moreover, the uniform electrostatic field created by the PDDA coating promotes a dense and consistent distribution of Zn ions, leading to uniform deposition patterns. These findings emphasize that even minor modifications, such as applying ultrathin polyelectrolyte layers, can lead to substantial improvements in electrodeposition processes. The implications of this research suggest that such cost-effective and straightforward methods could be widely applied to enhance the performance of Zn metal anodes.

## Supporting Information Available

Materials, instruments, methods, EIS analysis, elemental mappings, and measurement of electrodeposition by EDS (PDF)
