# Supplementary material for: Enhancing the Performance of Reversible Zn Deposition by Ultrathin Polyelectrolyte Coatings
Source: ACS Appl Mater Interfaces. 2023 Dec 2;15(49):57699–707. doi: 10.1021/acsami.3c14663 (PMC11156428; doi:10.1021/acsami.3c14663)
Supplement: Supplementary file 1 — am3c14663_si_001.pdf [file am3c14663_si_001.pdf]

Supporting Information

Enhancing the performance of reversible Zn deposition by ultrathin polyelectrolyte coatings

Netta Bruchiel-Spanier[*1], Omer Bluman[1], Linoy Lahav[1], Avigail Romem[1], Keren Shwartsman[1], Munseok S. Chae[2], Idan Bar-lev[1], Elad Gross[1], Netanel Shpigel[*3] and Daniel Sharon[*1]

1. Institute of Chemistry, The Hebrew University of Jerusalem, 9190401, Jerusalem, Israel
2. Department of Nanotechnology Engineering, Pukyong National University, Busan 48547, Republic of Korea
3. Department of Chemical Sciences, Ariel University, Ariel 40700, Israel.

Experimental:

Materials:

Zn and Cu foils, both 20 µm thickness, were purchased from Gelion. Zinc sulfate ($ZnSO_4$), sodium sulfate (Na_2SO_4), poly(diallyldimethylammonium chloride) (PDDA, 20 wt.% in water), polyethyleneimine (PEI, 50 wt. % in water), and Whatman A glass microfiber filter paper (Φ = 7/16 inch) were obtained from Sigma-Aldrich.

Instruments:

All electrochemical measurements were conducted using a VSP 300 station (Biologic) with a custom T-cell configuration. For both the Hydrogen evolution reaction (HER) and Tafel (corrosion test) measurements, a three-electrode T-cell was employed, with an Ag/AgCl (KCl 1M) reference electrode.

Zn deposition was characterized using an analytical high-resolution scanning electron microscope (Apreo 2S, Thermo fisher scientific). The properties of the ultrathin layer coatings were examined using contact angle measurements (Rame-Hart 100 goniometer, Rame-Hart instrument Co.) equipped with an automated dispensing system. Additionally, infrared reflection-absorption spectroscopy (IRRAS) analysis was performed using a reflection-adsorption cell (Harric, Inc.) in conjunction with an FTIR spectrometer (Vertex V70, Bruker). These measurements were carried out at room temperature under vacuum conditions, with parameters set consisting of 1,024 scans with a resolution of 4 cm^{-1} using a mercury-cadmium-telluride (MCT) detector.

X-ray diffraction data were obtained using Bruker D8 diffractometer with Cu Kα (λ = 1.5046 A) radiation source in the 2θ degree range from 5° to 90°. Thickness measurements of the self-assembled polyelectrolyte films on Cu-coated Si wafers were performed using an AFS-1(G3) multi-wavelength ellipsometer from Film Sense LLC. The average thickness was determined based on the values obtained from two separate samples.

Methods:

Sample preparation: Cu and Zn foils were cleaned with ethanol and deionized water for 5 min each using ultrasonication. Then, the foils were immersed in a 0.01 wt.% PDDA or PEI solution for 20 min with mild shaking at 50 strokes/min. Afterward, the foils were rinsed with deionized water and dried carefully. A fresh 1 M $ZnSO_4$ electrolyte was prepared for all tests by dissolving $ZnSO_4$ into deionized water.

Electrochemical measurements: Charge/discharge measurements were performed using a two-electrode T-cell configuration with a fixed current density of 0.5 mA/cm^2. The voltage was limited to between -1 V and 0.5 V (vs Zn^{2+}/Zn). A Wattman A filter paper was employed as a separator and 1 M $ZnSO_4$ aqueous solution was used as the electrolyte. This arrangement ensured constant pressure and a fixed distance between the working and the counter electrodes.

The symmetrical Zn||Zn cells were assembled with two bare or two coated Zn foils to test the reversibility. The Cu||Zn asymmetric cells were employed with bare or coated Cu foil as the working electrode and bare Zn as the counter electrode.

To evaluate the influence of the coating on corrosion inhibition, the polarization behaviour was analysed via linear sweep voltammetry (LSV) at a rate of 10 mV/cm in 1 M Na_2SO_4 aqueous electrolyte using three electrode cell which included both coated and uncoated Cu working electrodes, a high-surface-area carbon counter electrode, and an Ag/AgCl reference electrode. For Tafel measurements, bare and coated Zn were used as the working electrode and Ag/AgCl was used as a reference electrode. The surface area of the Zn electrode was fixed to 1.13 cm^2 using an electrode mask.

Chemical Characterization: Scanning electron microscopy was performed to investigate the surface morphology of the Zn deposits after depositing or stripping on a Cu foil. The samples were washed carefully washed with deionized water to remove residual electrolyte. Similarly, Samples for XRD measurements were prepared using

the same procedure. Rietveld analyses were performed for each sample using the powder profile refinement program[1], with the initial structural models adopted from The Materials Project[2] [2].

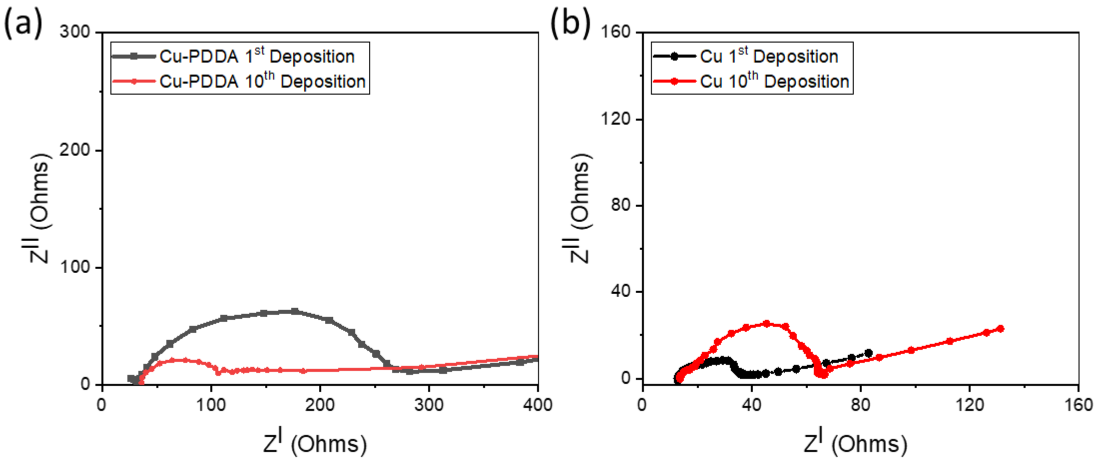

Figure S1: Electrochemical Impedance Spectroscopy (EIS) Analysis of Zn Deposition on (a) coated and (b) uncoated Cu ssubstrates after the first and tenth electrodeposition process.

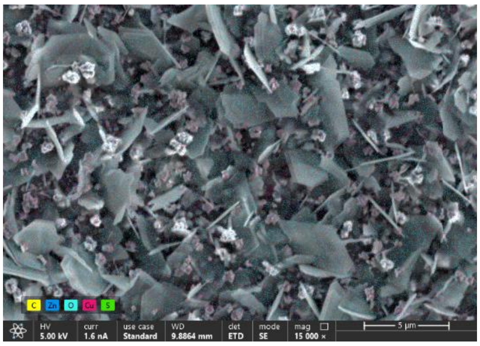

Element	Atomic %	Atomic % Error
C	1.8	0.1
S	4.1	1.1
Zn	33.8	2.8
O	35.2	8.5
Cu	19.2	7.7

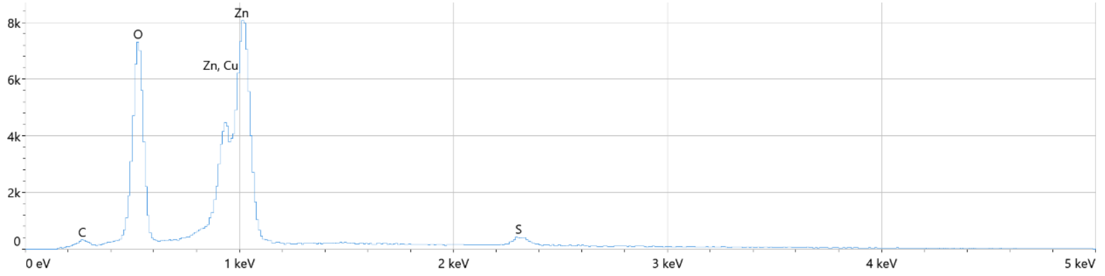

Figure S2: Elemental mapping and content of bare-Cu substrate after electrodeposition measured by EDS.

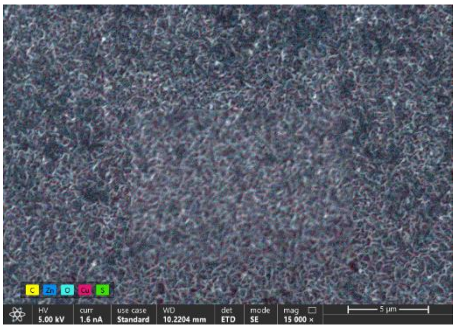

Element	Atomic %	Atomic % Error
C	2.55	0.3
S	3.1	0.1
Zn	44.55	3.45
O	25.7	2.4
Cu	24.1	1.4

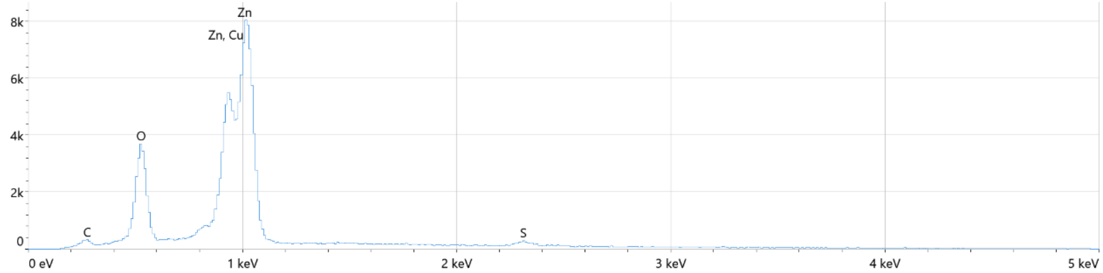

Figure S3: Elemental mapping and content of Cu-PDDA substrate after electrodeposition measured by EDS.

References:

(1) Toby, B. H. EXPGUI, a Graphical User Interface for GSAS. *urn:issn:0021-8898* **2001**, *34* (2), 210–213. https://doi.org/10.1107/S0021889801002242.

(2) Jain, A.; Ong, S. P.; Hautier, G.; Chen, W.; Richards, W. D.; Dacek, S.; Cholia, S.; Gunter, D.; Skinner, D.; Ceder, G.; Persson, K. A. Commentary: The Materials Project: A Materials Genome Approach to Accelerating Materials Innovation. *APL Mater* **2013**, *1* (1), 011002. https://doi.org/10.1063/1.4812323.
